# Contradicting habitat type-extinction risk relationships between living and fossil amphibians

**DOI:** 10.1098/rsos.170051

**Published:** 2017-05-10

**Authors:** Melanie Tietje, Mark-Oliver Rödel

**Affiliations:** 1 Museum für Naturkunde, Leibniz Institute for Evolution and Biodiversity Science, Invalidenstraße 43, 10115 Berlin, Germany; 2 Berlin-Brandenburg Institute of Advanced Biodiversity Research (BBIB), Berlin, Germany

**Keywords:** Amphibia, fossil record, extinction risk, habitat trait, Anthropocene

## Abstract

Trait analysis has become a crucial tool for assessing the extinction risk of species. While some extinction risk-trait relationships have been often identical between different living taxa, a temporal comparison of fossil taxa with related current taxa was rarely considered. However, we argue that it is important to know if extinction risk-trait relations are constant or changing over time. Herein we investigated the influence of habitat type on the persistence length of amphibian species. Living amphibians are regarded as the most threatened group of terrestrial vertebrates and thus of high interest to conservationists. Species from different habitat types show differences in extinction risk, i.e. species depending on flowing waters being more threatened than those breeding in stagnant sites. After assessing the quality of the available amphibian fossil data, we show that today's habitat type-extinction risk relationship is reversed compared to fossil amphibians, former taxa persisting longer when living in rivers and streams, thus suggesting a change of effect direction of this trait. Neither differences between amphibian orders nor environmentally caused preservation effects could explain this pattern. We argue this change to be most likely a result of anthropogenic influence, which turned a once favourable strategy into a disadvantage.

## 1. Introduction

With an increasing number of species being potentially threatened by anthropogenic environmental changes, scientists have been searching for new insights into how to determine the extinction risk of species [1–3]. Using traits—biotic and spatial characteristics of a species—has become a common method to estimate this risk [4,5]. The International Union for the Conservation of Nature (IUCN) for example uses a set of trait categories to assess the risk of species for their Red List, which is a prime guide for conservation strategies [6].

When traits for a large variety of species are applied toward predictive conservation, it would be important to know that the effect of a certain trait is actually stable, or at least influences the extinction risk of different species in the same direction. Hence, it is of importance to test whether the targeted traits are valid in extinction risk assessments across different species and over longer time scales. Such validation would make the assessment process easier and comparable across a larger number of different taxa. For instance a widely applied trait is species' geographical range size [7]. Various examples from living and fossil species revealed geographical range size as a good proxy for extinction risk [8–10].

However, it has also been shown that some traits, like body size and life history, do not necessarily have the same effect size or even direction for differing taxa [8,11,12]. Given that differences in the response of certain traits are possible between taxa that exist today, the validity of traits over longer time scales should be of equal importance as the current state of species is always a product of their evolutionary past, and traits naturally evolve together with their species. Assessing the trait-extinction risk relationship in the past is possible by using the fossil record of species, which provides a great archive of multitudinous species with different trait combinations over various timespans [13,14]. Not all, but some of their traits, i.e. morphological ones, have been preserved over long time periods, and these can be combined with the species' longevity (duration). Thus, we can follow species from (an approximate) first to last appearance in the fossil record and are able to test for a correlation between certain traits and species' durations, consequently drawing inferences about the influence of traits on the extinction risk.

In this study, we intended to assess the impact of habitat traits on amphibian survival in the past. We chose to use amphibians for this task for several reasons: (i) amphibians are an old taxonomic class, reaching 370 million years back into the late Devonian [15,16]. This increases the chance to gather fossil species over a broad time scale; (ii) most amphibians have aquatic ontogenetic stages [17], which makes them dependent on bodies of water and should be beneficial for fossil preservation; (iii) current amphibian species are almost completely assessed for their conservation status and comprise the highest proportion of endangered species among terrestrial vertebrates [18,19] and thus are of high interest in current conservation efforts; and (iv) they exhibit a distinct difference in proportion of endangered species between lotic (flowing) and lentic (stagnant) freshwater habitats, lotic species being seemingly more threatened [20]. This habitat information is preserved in the fossil record.

We herein investigated whether an increased extinction risk in lotic amphibian species can be as well inferred from the fossil record. Based on data from two databases and further literature, we assessed the duration of extinct species in different habitat types. We tested for phylogenetic influence as well as for influence of habitat-dependent preservation bias. Before analysing our data, we evaluated the quality of the amphibian fossil record by applying several preservation and completeness metrics.

## 2. Methods

### 2.1. Data sources

The fossil amphibian data were collected from the Paleobiology Database (PbDb) on 15th February 2016 using http://fossilworks.org [21] and from the FosFARbase on 18th July 2016 using http://www.wahre-staerke.com [22], collecting all available amphibian data with no restrictions on geological time. Data downloaded included lithology, location and time (geological stage) of the fossil occurrences. Lithology data was solely available from the PbDb and complemented by literature research where possible (see supplement for lithology data with references). Downloads followed keyword searches with ‘Allocaudata, Amphibia, Anura, Caudata, Gymnophiona, Temnospondyli, Urodela’, and thus comprised amphibian orders including their stem groups (Salientia, Urodela and Parabatrachia), Lepospondyli, and the group of temnospondyl amphibians. Caudata and Urodela were used as interchangeable terms owing to differing taxonomic opinions of the two databases. By including Lepospondyli and Temnospondyli, we account for both the temnospondyl and lepospondyl origin hypothesis of modern amphibians [23]. Our exact usage of taxonomic terms is explained in the electronic supplementary material, table S1. Extinct species' names follow the taxonomy used in the PbDb. Taxonomy from fosFARbase was adapted to the names used in PbDb (electronic supplementary material, table S2). Duplicate records were manually excluded from the search results as were all species or genera marked with ‘aff.’, ‘cf.’ or ‘?’. We identified extant species according to their presence in the ‘Amphibian species of the World’ database [24] and excluded those species from the fossil list of taxa.

The final dataset contained 1658 occurrences from 620 extinct species, covering the amphibian fossil record from the Carboniferous to the Holocene. In this dataset, 816 occurrences from 358 species had lithology information and thus were included in the analyses of habitat influence on species duration. For comparison, we collected taxon lists for extant and fossil mammals from the PbDb (10th February 2016).

### 2.2. Data quality and completeness

As, for obvious reasons, data from the fossil record will be always incomplete, it is of high importance to test datasets for potential preservation flaws [25,26]. As information on the quality of the amphibian fossil record is sparse, we first assessed quality and completeness of our data by checking four factors that could potentially bias our results: (i) preservation potential of amphibians; (ii) completeness of the fossil record; (iii) reliability of single-interval species; and (iv) preservation differences between habitats.

The preservation potential was assessed by estimating the proportion of living taxa with a fossil record and by calculating the preservation probability. The proportion of living taxa with a fossil record shows the basic potential of a group to be preserved by counting how many living taxa of that group are already preserved as fossils [27]. Another preservation probability estimate uses the range-frequency distribution of fossil taxa. Using equation (2.1), following Foote and Raup [28] and Foote and Sepkoski [29], we calculated the preservation probability for amphibian species, genera and families:

2.1 $$\text{preservation probability}=\frac{f_{2}^{2}}{f_{1}*f_{3}}.$$

Species duration was measured as number of geological stages from first to last occurrence of the species. The frequencies *f*_1_, *f*_2_ and *f*_3_ represent the proportion of taxa with durations of one, two and three geological stages. Lower ratios indicate more taxa with short ranges and therefore a lower preservation probability. Completeness of the fossil record was checked by applying the simple completeness metric (SCM, [30]). The SCM measures completeness of the fossil record based on the gaps in each taxon's record. It was calculated as the ratio of observed fossil occurrences to total inferred fossil occurrences ([31], equation (2.2)). As result, lower SCMs show fewer gaps in the fossil record and therefore a higher completeness. Calculations were done on species, genus and family levels:

2.2 $$\text{SCM}=\frac{\text{known record}}{\text{assumed record}}.$$

As the fossil data included a high percentage of single-interval species, simply removing them would have resulted in a huge loss of diversity and the loss of potentially very shortly lived species. We thus investigated the reliability of short durations in these species following Fitzgerald and Carlson [32]. We determined the proportion of single-interval species from lagerstätten as well as monographic effects in our data to test for their effect on the number of single-interval taxa. Lagerstätten provide an exceptional amount and/or quality of preserved fossils [33], increasing the probability to find rare species. Similarly a focused sampling on one temporal interval can cause an increase in single-interval taxa (monographic effect), as it increases the probability to find rare species. Both factors intensify sampling of one region or time frame and can lead to a higher proportion of single-interval taxa. Rare species detected this way would probably be overlooked with less intense sampling, which would result in these species being falsely detected as single-interval species. We assessed the proportion of single-interval taxa described in monographies (publications which covered more than 20 occurrences) and compared those to the proportion in the complete dataset. Different proportions of single-interval taxa would indicate that the data suffer from monographic effects. We estimated the influence of lagerstätten by comparing the amount of single-interval species from lagerstätten and the complete dataset using Pearson's *χ*^2^ test for count data (significance level *α* = 0.05). Besides lagerstätten and monographic effects, we also tested for correlation between geological stage durations and species richness, number and proportion of single-interval species per stage. A correlation could indicate that single-interval species were not necessarily short-lived, but a result of the temporal resolution of the rock record, meaning the variability of stage durations might influence the amount of single-interval species. Correlation analyses were done using Spearman's rank correlation.

Finally, differences in preservation probability between habitats were assessed using the specimen completeness metric by Benton [34]. This metric assigns each occurrence increasing values from one to five, according to the conditions of the available specimens (isolated bones, one (nearly) complete skull, several skulls, one (nearly) complete skeleton, and several skeletons). High values indicate a good preservation of the specimens, and differences in specimen completeness between habitats were calculated. Differences between taxonomic groups were also taken into account. Finding more complete specimens in a particular habitat would indicate a higher preservation probability and thus longer overall species durations. As the condition of specimens was not available for all data, respective samples of occurrences from each environment were used (*n* = 81, 72, 35, 14 for *stagnant*, *low-velocity*, *medium-velocity*, *high-velocity*, respectively). The specimen completeness between habitats was compared using Kruskal–Wallis rank sum test and Wilcoxon rank sum test for pairwise comparisons (fdr *p*-value correction).

### 2.3. Species duration, habitat and range size

Species durations were determined following Harnik [8]. We calculated the distance between geological stage mid-points of first and last occurrence of a species in millions of years, rounded to the next 1 million years. Species durations were compared between different taxonomic groups and habitats, as well as between habitats within each taxonomic group to control for phylogenetic dependence, using Kruskal–Wallis rank sum test and Wilcoxon rank sum test for pairwise comparisons (fdr *p*-value correction). To quantify the difference between those groups, we compared trimmed means. The trimmed mean is a robust estimate for the mean of an asymmetric distribution [35]. We used default settings provided by the describe function from the R psych package (trim = 0.1).

**Figure 1.**
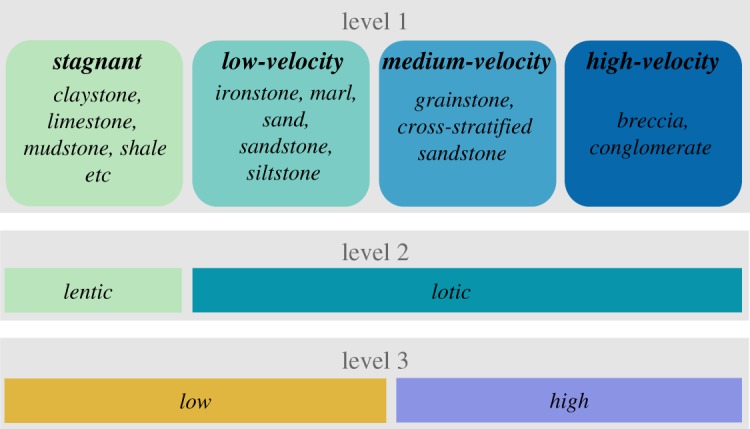
Lithologies were assigned to four habitat categories (*stagnant*, *low-velocity*, *medium-velocity* and *high-velocity*), reflecting an increase of water flow energy and thus a continuum from stagnant to strongly flowing water, which is also visible by an increase in grain size and sedimentary structures (e.g. cross-stratification). Other habitat categories used in the analysis were *low* and *high* energy (level 3), reflecting a broader categorization scheme, and the two contrasting habitat categories *lentic* and *lotic* (level 2). Further rock types representing the category *stagnant* are: coal, diatomite, dolomite, gyps lignite, marl, peal, phosphorite and tuff.

Fossil occurrences were assigned to different habitat categories based on their lithological context. As lithology reflects the sedimentary environment, and therefore the habitat in which the organism fossilized [36], we used lithology as a first order approximation of energetic regime and assigned each occurrence to one of four basic habitat categories. The assignment process is depicted in figure 1. We used three different sets (levels) of habitat categories: the first level comprised four distinct habitat categories, representing increasing energy in each depositional setting (*stagnant, low-velocity, medium-velocity, high-velocity*); a second level differentiated *lentic* from *lotic* depositional setting; and a third level indicated either a *low* or *high* energetic depositional setting. Level two and three assignments derived from level one data. We avoided redundant assignments and therefore bias caused by differing number of occurrences between species. When comparing species duration between habitats, the duration of a species got assigned once to the same habitat category on each level, regardless of the number of lithologies it occurred in. Therefore, a species with occurrences e.g. in claystone and shale was included only once in the category *stagnant*. The same principle was followed for the other habitat categories. Occurrences were excluded from analysis if they did not provide sufficient information to allow assignment to one of the four first level categories. This was the case for records with missing lithology data or data entries that did not allow inference on original energetic setting (e.g. ‘cave infill’). Differences in durations between habitat groups were tested using Kruskal–Wallis rank sum test and Wilcoxon rank sum test for pairwise comparisons (fdr *p*-value correction).

We aimed at excluding phylogenetic dependence from purely environmental effects and therefore checked for differences between the durations and habitat preferences of the taxonomic groups. If species within some taxa naturally would have had longer/shorter durations than those of other taxa, and in addition would only occur in certain habitats, then longer durations in these habitats might not be caused by habitat, but simply be a result of one taxon dominating the habitat. A chi-squared test was used to test for differences between the expected and the observed frequencies of habitat categories within taxa. Differences in duration between habitat categories were compared within taxonomic groups.

Comparisons between taxonomic groups were always done on two taxonomic levels to account for the phylogenetic structure of the data, making sure to only compare taxa of the same hierarchical level. As Lepospondyli and Temnospondyli are both potential stem-group candidates for lissamphibians, we did comparisons between Lissamphibia groups (Allocaudata, Gymnophiona, Salientia and Urodela) and between the higher groups Temnospondyli, No-Temnospondyli (all species except Temnopondyli), Lepospondyli and No-Lepospondyli.

Geographical range size was included in our analysis as range size is a well-known influential factor for extinction risk in extinct and extant species [37,38]. Range size calculations followed the approach by Finnegan *et al*. [3] based on occupancy. A grid of 2 × 2 decimal degrees was projected on the palaeocoordinates of all occurrences and each occurrence assigned to a grid cell ID. The number of different grid cells, occupied by one species, resulted in the final grid cell count for each species. Geographical ranges were compared in the same way duration was compared between different habitat groups.

All analysis were done using the R environment v. 3.3.2 [39] with the additional packages ggplot2, readxl, gridExtra, psych, reshape and gsubfn [40–45]. An R script including analysis can be found in the electronic supplementary material.

## 3. Results

### 3.1. Quality and completeness

Our dataset comprises fossil amphibian occurrences from the Visean to the Holocene. Median geological stage duration was 5.7 million years with a median average deviation of 2.5 million years (*n *= 53).

The proportion of living amphibian families with a known fossil record was 33%, which is rather low compared to various groups of invertebrates and fishes (electronic supplementary material, figure S1). However, comparing amphibians with mammals showed that the preservation potential was smaller for amphibian families, but higher for amphibian genera and species (electronic supplementary material, table S3). The preservation probability of amphibians based on the duration frequency distribution showed that 33% of the amphibian species and about half of the genera were preserved at least once from one geological stage to the other (electronic supplementary material, table S3).

**Figure 2.**
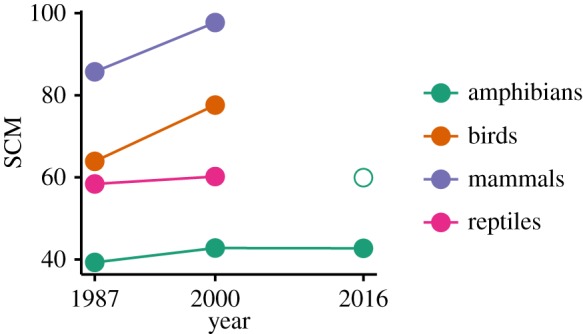
Comparison of simple completeness metric (SCM) for Cretaceous tetrapod groups from different years. Adapted from Fara & Benton [46] and completed with SCM values based on our dataset for 2016. SCM was calculated on the family level. The empty circle represents the total SCM value for amphibians; the solid ones give the value for Cretaceous families only.

The SCM for species, genera and families showed values between 0.60 and 0.94 (electronic supplementary material, table S3). A comparison of Cretaceous amphibians with other vertebrate taxa placed their SCM at the lower end, yet slightly improving over time (figure 2, [46]). The SCM indicated that from all geological stages potentially containing amphibian fossils, at least 60% actually did contain fossil occurrences.

While testing for potential biases concerning the durations of species, we found no correlation of stage duration with species richness, number of single-interval species, or proportion of single-interval species (|*ρ*| < 0.01, *p *> 0.95, *n *= 53), indicating that geological stage duration had no biasing effect on the number of single-interval species. Ten per cent of PbDb species comprised occurrences tagged as coming from lagerstätten. Of those species, 69% were single-interval species, compared to 87% of single-interval species in the remaining, non-lagerstätten, dataset. Proportions of single-interval species thus differed significantly ($\chi_{1}^{2}=7.9$, *p *< 0.01), but were not biased towards lagerstätten. In addition there was no evidence for monographic effects, as publications describing a larger number of occurrences (more than 20) contained only 20% single-interval species in these occurrences. Lagerstätten and monographic effects were solely assessed for PbDb data, however we did not expect the two databases to differ in this regard as proportions of single-interval species occurrences were within comparative ranges in FosFARbase and PbDb (38% and 51%, respectively).

By comparing specimen completeness between different habitat categories we tested for habitat-dependent preservation potentials and for differences in this potential between taxonomic groups. For all amphibians, we found significant differences between the *stagnant* habitat category with both *low-velocity* and *medium-velocity* categories (*p *< 0.05), with a larger trimmed mean in *stagnant* (electronic supplementary material, table S4). The results were similar when we tested for differences individually within the groups Urodela, No-Lepospondyli, No-Temnospondyli and Temnospondyli. Specimen completeness also differed between the larger taxonomic groups, but not between lissamphibian groups (electronic supplementary material, table S5). Temnospondyli and Lepospondyli showed higher trimmed mean specimen completeness than other taxonomic groups. These results suggest a habitat influence, and, to some degree, a taxonomic influence on specimen completeness, with specimens from low-energy environments being more likely to be documented in the fossil record than specimens living in high-energy environments.

Based on these analyses on the quality and completeness of the fossil data, we are confident to use our data for the following analyses on differences in species' durations between habitats.

### 3.2. Habitat

**Figure 3.**
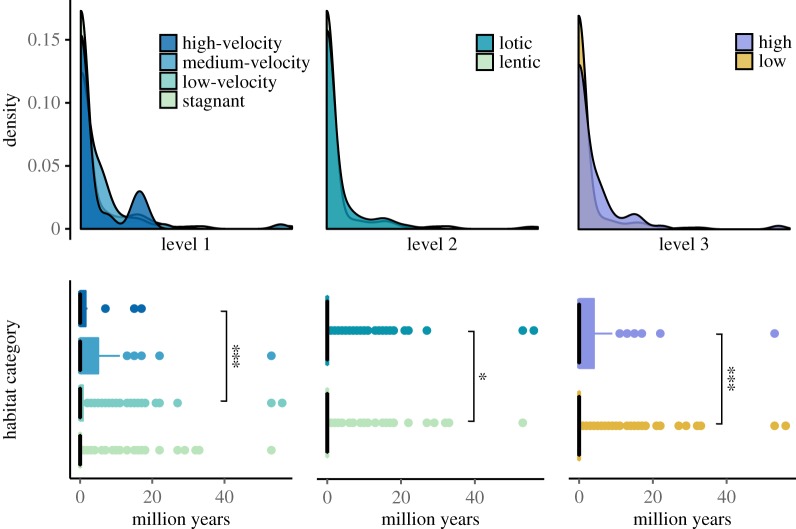
Durations of amphibian species in different environments. Species were grouped into four basic (level 1) and two broader environmental categories (level 2 and 3; compare figure 1). Sample sizes for groups were: *stagnant* (214), *low-velocity* (130), *medium-velocity* (56) and *high-velocity* (18); *lentic* (216) and *lotic* (176); *low* (319) and *high* (71). The upper panel shows the density distribution of durations (bandwidth = 2 million years), the lower panel shows the durations as boxplots, with black lines indicating the median and coloured areas illustrating the range between first and third quartiles. Significant differences are indicated by one, two and three asterisks indicating *p*-values smaller than 0.05, 0.01 and 0.001, respectively (for exact values compare electronic supplementary material, table S6). The largest outliers were caused by *Scapherpeton tectum* and *Gobiops desertus*, two extremely long living species.

Lithological information was available for 816 occurrences from 358 species. We investigated if durations of these species were connected to habitat type on three different levels (as defined in figure 1). On the first level, we found significant differences in species' durations among habitat categories (electronic supplementary material, table S6). Pairwise comparisons showed that species' durations in *medium-velocity* were longer than those from *stagnant* depositions (*p *< 0.001; figure 3; electronic supplementary material, table S6). Comparing durations from the broader categories *low/high* as well as *lentic/lotic* levels, showed *high* and *lotic* species to have longer durations than species from *low* or *lentic* settings (*p *< 0.05, figure 3, electronic supplementary material, table S6). These results indicate that species which lived in high-energy environments prevailed for longer periods than species from low-energy environments.

**Figure 4.**
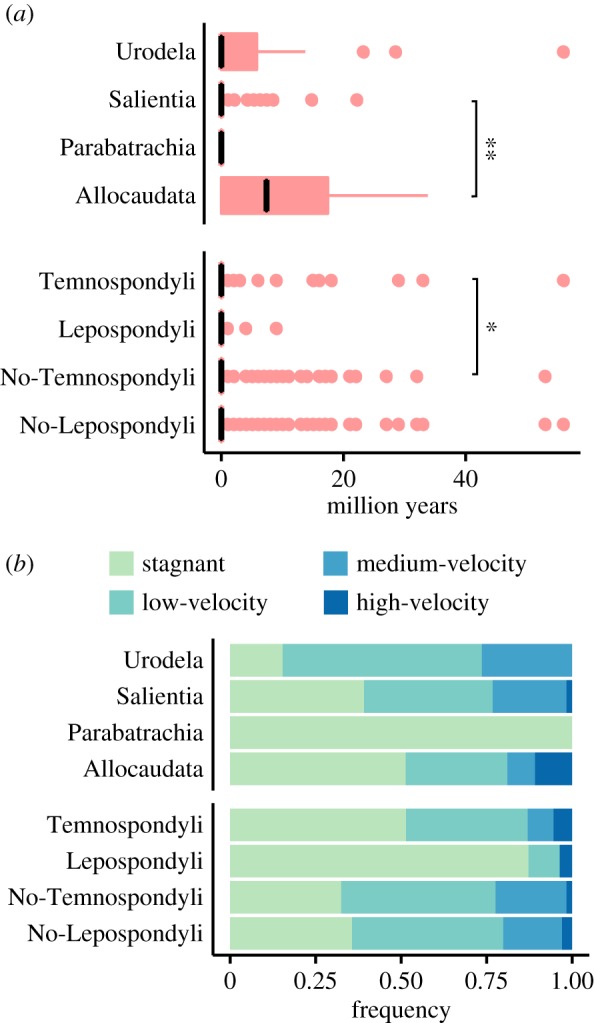
Durations and habitat preferences. (*a*) Durations of amphibian species from different taxonomic groups. Numbers of species for the groups were Allocaudata (11), Urodela (39), Parabatrachia (2), Salientia (80), No-Temnospondyli (171), No-Lepospondyli (310), Lepospondyli (39), and Temnospondyli (178). Black lines in boxplots indicate the median and coloured areas illustrate the range between first and third quartiles. Significance differences are depicted by one, two and three asterisks indicating *p*-values smaller than 0.05, 0.01 and 0.001, respectively. (*b*) Frequency of species in habitat categories for different amphibian groups, each species counted once per habitat category. Habitat categories as defined in figure 1. Parabatrachia had just two occurrences (from two species) which were from the same, *stagnant*, environment.

We tested for a phylogenetic signal in our data by comparing durations between taxonomic groups as well as between habitat preferences. We found significant differences between durations of Allocaudata and Salientia, with Allocaudata displaying longer median durations. Significant differences were also found between Temnospondyli and all other taxa (No-Temnospondyli), with No-Temnospondyli having a larger trimmed mean duration (figure 4*a*; electronic supplementary material, table S7).

For habitat preference, expected and observed frequencies of habitat categories did not significantly differ among lissamphibian groups, but among the taxonomic groups Lepospondyli, Temnospondyli, No-Lepospondyli and No-Temnospondyli ($\chi_{9}^{2}=30.565$, *p <* 0.001, figure 4*b*). Therefore, taxonomic identity and habitat usage were not entirely independent. Comparing the observed habitat frequencies with the expected frequencies revealed more than randomly expected occurrences of Lepospondyli in *stagnant* and less in *low-velocity* and *medium-velocity* habitats (electronic supplementary material, table S8).

As there were differences in the species duration, and to some extent in the habitat category frequencies between taxonomic groups, we checked for taxonomic influence in our observed duration pattern. We analysed the durations in habitat categories in all taxonomic groups individually. We detected significant duration differences among habitats for the groups Salientia, No-Temnospondyli and No-Lepospondyli on all environmental levels (except No-Lepospondyli in level 2, electronic supplementary material, table S9). Pairwise comparison for level 1 habitat categories revealed significant differences in the comparison groups *stagnant/low-velocity* and *stagnant/medium-velocity* for all three taxonomic groups (except No-Lepospondyli *stagnant/low-velocity*, electronic supplementary material, table S10), with *low-velocity* and *medium-velocity* showing 1.1 to 3.5 million years larger trimmed mean durations compared to *stagnant* habitat. *Lotic* species had 1.1 to 1.9 million year longer trimmed mean durations than *lentic* species, and trimmed mean durations for *high-velocity* species were 1.3 to 1.8 million years longer than for *low-velocity* species. These results indicate that the pattern of longer durations in high-energy environments was strongest in Salientia, with Urodela and Temnospondyli showing a similar but non-significant trend.

While determining if geographical range might influence our results, we found significant differences between low and high-energy habitat categories on levels 1 and 3 (*p *< 0.001), with trimmed mean geographical ranges being larger in high-energy environments (electronic supplementary material, figure S2, table S11). The results showed that species from low energetic environments had a smaller geographical range size than species from high energetic environments. We observed similar results when controlling for geographical range size by analysing species with small and large range separately (electronic supplementary material, table S12).

Although we assume that most single-interval species in our data represent a real signal rather than being a result of preservation bias, we still wanted to consider the potential influence of false single-interval species. Therefore, we checked for equal distribution of single-interval species among habitats and tested for duration differences among habitats without single-interval taxa. Pearson's *χ*^2^ test showed that proportions of single-interval species differed between habitat groups ($\chi_{3}^{2}=15.9$, *p *< 0.01), with *stagnant* showing more and *medium-velocity* showing less single-interval species than expected by chance (electronic supplementary material, figure S3, table S13). On level 3, habitat differences were significant as well ($\chi_{1}^{2}=16.2$, *p *< 0.001) with 87% of low energetic setting species being single-interval species compared to 66% in high energetic settings (electronic supplementary material, figure S3). Level 2 showed no significant habitat differences for single-interval species. We therefore concluded that single-interval species did overall occur more often in *low* energetic habitats. Excluding single-interval species from the duration pattern analysis among different habitats resulted in severe data reduction and the loss of the pattern observed in the complete dataset (electronic supplementary material, figure S4, table S14).

## 4. Discussion

Our analyses showed that extinct amphibian species differed in their duration depending on the habitat they lived in. Contrary to todays' situation, where extinction risk seems to be higher in species living and breeding in lotic waters [20], we found that extinct species persisted longer through time when they occurred in flowing water. An extensive quality analysis of our data not only revealed that amphibian data are at the lower end of available vertebrate fossil record quality (see also [30]), but also confirmed that the data were of sufficient reliability for our analyses. In particular these tests proofed that the most critical part of our data, the single-interval taxa, could be used as done herein.

### 4.1. Quality and completeness

Without knowing the weak spots of palaeontological data, the nonetheless fragmentary fossil record can easily lead to false inferences. Although we are well aware that the fossil record of amphibians is not perfect, we argue that for our approach the available data were sufficient.

To our knowledge the only studies hitherto explicitly examining the quality of the amphibian fossil record applied an SCM and showed a record with more gaps than mammals, birds and reptiles in the Cretaceous period [30,46]. While our analysis confirmed these findings, we also found that the SCM for all time intervals was higher than in the Cretaceous (electronic supplementary material, table S3), which is also true for the complete tetrapod fossil record (fig. 2 in [30]). However, the SCM seems to be heavily influenced by the high number of single-interval taxa, naturally not containing any time gaps, and therefore potentially biasing the metric. Calculating the SCM on durations without their range endpoints gave much lower metric values (electronic supplementary material, table S3), which confirms the influence of single-interval species. The SCM was initially developed for usage on the family level [30]. However, we worked on the species level, and thus we considered testing the reliability of single-interval species to be more important than the SCM.

The preservation probability suggests the amphibian fossil record to be comparable to that of corals (electronic supplementary material, figure S1), which are often used for community structure analysis [47–49]. But comparing a terrestrial vertebrate taxon to a marine invertebrate taxon might not be very insightful owing to the usually much better preservation in marine sediments [50] and the vastly differing lifestyles of the taxa. The comparison of the amphibian data with those from mammals, being predominantly terrestrial vertebrates as well, instead showed the preservation potential of the amphibians to be lower on the family, but higher on the genus and the species levels (electronic supplementary material, table S3). This imbalance in preservation potential between taxon levels could hint at a family recognition problem, as morphological character differences between families are often subtle in amphibians and might have been lost owing to their mostly fragile nature. However, this does not affect our analysis as we solely focused on species durations.

More worrying was the high percentage of single-interval species in our fossil data, whose exclusion resulted in the loss of any significant difference in duration between habitats (electronic supplementary material, figure S4). Our analysis following Fitzgerald and Carlson [32] showed that the single-interval species are probably a real phenomenon and not produced by preservation or sampling biases. Dubey and Shine [51] support this view by giving a median age for extant anuran species of 1.5 million years, which is smaller than the length of most geological stages, and making it plausible for many species to be present in only one time interval. We also saw single-interval species being more common in low-energy habitats (electronic supplementary material, figure S3), which we assume to be a real signal, as we would expect the contrary given the better preservation potential in these environments. We argue that for our dataset the resulting loss in biodiversity and sample size would pose a greater bias to the results than occasional false single-interval species would do.

In accordance with our initial assumption of preservation potential being higher in calmer environments [36], we observed the highest specimen completeness in lentic habitats. This suggests that species from high-energy habitats should be affected the most by preservation bias and might have truncated durations as a result. As our high-energy records actually had longer durations, we conclude that the observed duration pattern is not because of a preservation bias. On the contrary, the effect of habitat on species duration might be even stronger than observed. On a taxonomic level, the higher overall specimen completeness detected for Temnospondyli and Lepospondyli is most likely attributed to the more robust morphology of Temnospondyli [52], and the preference of lentic water bodies by Lepospondyli (figure 4*b*). However, the higher preservation potential did not result in longer durations for Temnospondyli (figure 4). We initially assumed that good preservation results in potentially longer durations of species. In addition, differences in preservation potential of habitats might not only act on the duration but also on the total number of preserved species. An increase in preservation potential, like in low-energy habitats, might result in a larger proportion of short duration species, as fragile and rare species become more likely to be preserved in the first place. On the other hand, the preservation of all other species equally becomes more likely, and therefore their durations potentially longer. Depending on the ratio of newly added short duration species on one end and extended species durations on the other, a higher preservation potential might result in longer or shorter overall durations, or even no change at all. How many rare and fragile species become added to the preserved fauna with increasing preservation potential might depend on the morphological characteristics as well as abundance distribution of the species within the respective fauna. As clarification of this issue is beyond the scope of this study, we conservatively assume the effect of changing preservation potential between habitats to be neutral.

### 4.2. Habitat and species’ duration

A basic assumption for our study is the correct assignment of occurrences to habitat categories. Out-of-habitat transportation might be a factor influencing our outcomes, as post-mortem transportation could result in the species being assigned to the wrong habitat category. However, in a review on the quality of the fossil record Kidwell & Flessa [53] state that out-of-habitat transportation by secondary deposition is unlikely to occur. We have to admit, that we cannot judge what might be the more common scenario, dead animals washed from streams into ponds and lakes, or the converse way. Anyhow, based on the above review results and various biological data for many of our species, we are confident that our habitat categories reflect the actual habitats of the respective species.

More important for the interpretation of our main result, longer durations of species in high-energy habitats, indicating a lower extinction risk compared to their relatives in low-energy habitats, might be biases by phylogeny or other traits.

However, the phylogenetic influence on duration differences between habitats turned out to be rather small. Durations differed between few taxonomic groups (Temnospondyli and Allocaudata, figure 4*a*), which we expect to be of no influence to our main result as both groups did not differ in their habitat preference. The only group with a habitat preference that differed from the other taxa (Lepospondyli, figure 4*b*) showed again no difference in its duration pattern. Therefore, differences in duration between taxonomic groups did not co-occur with differences in the habitat preference of these groups. We also found the same duration differences between habitats in all except two taxonomic groups alone, which supports the phylogenetic independence of the results. In the two groups in which this trend was not statistically significant, Lepospondyli and Allocaudata (electronic supplementary material, table S9), we attribute the lack of any pattern to the low sample size in general and especially in high-energy environments for Lepospondyli.

A different trait influencing our results could be the geographical range size of species, which we found to have a positive correlation with the flow energy of species' habitats (electronic supplementary material, figure S2). As geographical range size is widely acknowledged as an important factor for extinction risk in amphibians and other taxa [38,54,55], this finding supports the lower extinction risk in high-energy habitats. However, controlling for geographical range size did not change the observed duration pattern between habitats, therefore it cannot be the only cause for our results. This result contrasts several findings from studies on insects with aquatic stages, showing that lotic species have smaller ranges than lentic species [56,57]. This is attributed to the lower temporal stability of stagnant water bodies and the resulting higher dispersal ability of inhabiting species [58]. One could argue that our observed larger ranges in high-energy habitats are the result of increased dispersal ability, caused by a widely spread water body with potentially active transportation. Another possible explanation for the reversed range size pattern between habitats might be the larger body sizes of early amphibians (Temnospondyli), which made species less prone to predation and thus able to inhabit larger, high-order streams, as well as possibly enabled them to simply move longer distances than smaller species.

In accordance with the larger range sizes in lentic species today (see above), it is usually assumed that amphibian species using low-energy habitats are less prone to extinction. Species breeding in ponds, for example, have to cope with lower habitat stability [58], which might make them more tolerant to environmental fluctuations. Higher extinction risk in lotic habitat species today [20] might be further caused by their mainly mountainous distribution, which naturally restricts their range size and isolates them from other areas with matching environmental conditions. It was also suggested that species associated with rivers might be more exposed to diseases [20].

To explain why a habitat type apparently changed from beneficial to detrimental for long species survival, one has to consider (i) changes in the habitat demands of amphibians over time, and (ii) changes in the habitat itself.

Habitat demands of a species are defined by various traits, for example morphology and life history. The most obvious change in morphology during amphibian evolution might be the overall decrease in body size. Comparing the 6 m length of the largest Temnospondyli with amphibians today [17], the latter are much smaller (the by far largest being *Andrias davidianus* with 1.5 m total length). However, current anuran species are not smaller than their ancestors, but still showed the inversed pattern in extinction risk between habitats. Therefore, the decrease in overall body size seems to be at least not the main reason for the change. The most prominent amphibian life-history trait, the biphasic life cycle with aquatic larvae, is assumed to be the ancient state for lissamphibians and supported by Temnospondyli fossils [23]. Moreover, the even more complex anuran metamorphosis with the apomorphic tadpole has been recorded since the mid Jurassic [59]. If we otherwise assume that a shift in life history from aquatic to more terrestrial lifestyles in the amphibian evolution might have caused the difference in duration pattern between past and present, we would expect to observe a difference between amphibian orders, as these differ in their lifestyles, too. However, in our data, species across all orders displayed a higher extinction risk in lotic habitats.

If habitat demands of amphibians have remained basically unchanged, then changes in the habitat itself could be a reason for the observed differences in habitat-extinction risk relationship. It is thus tempting to assume an anthropogenic influence on that relationship, as anthropogenic effects on the environment clearly were not present in the fossil past. More specifically, there are several examples of anthropogenic factors influencing the amphibian fauna associated with rivers. Most importantly altered river structures and communities are an important factor that negatively affects amphibians. In various regions natural river systems, and in particular the abandoned channels and regularly flooded areas, are severely declining. On the other hand, exotic fish species have been released worldwide, potentially spreading diseases and increasing predatory pressure on tadpoles and adults [60]. Further logging activity along rivers has an indirect effect on the physical characteristics and the macroinvertebrate community of streams [61], which probably affects the amphibian community as well. More generally, disturbance of the habitat as measured by the amount of forested, agriculturally or residentially used area and concomitant alterations in water temperature, pH and dissolved oxygen have a negative influence on the relative abundance of stream-dwelling salamander populations [62]. These influences might act stronger on river habitats, which were and are of great importance to humans [63] and therefore strongly influenced [64]. Although we admit this to be speculative, we assume that most lotic species experienced more stable environmental conditions than their relatives living in lentic, often temporary habitats. The latter might be thus naturally already better adapted to frequent habitat changes. Lotic species in contrast might be particularly at risk by a multitude and increasing human-induced environmental changes, and the advantageous stability of this habitat over longer geological time scales has consequently been reversed.

## 5. Conclusion

We detected increased extinction risk in fossil amphibian species from low-energy water habitats, which objects today's situation. A trait character once favourable for a species turned into a disadvantage. Given that a likely reason is altered habitat conditions via anthropogenic influence, our work shows that the trait-environment interaction is an important factor to consider when learning about the influence of traits from the past. The fossil record might provide us with sort of a baseline, ancestral extinction risk, which obviously does not consider human influence. The differences between expected and observed influence on extinction risk might give us an insight about the underlying mechanisms of complex traits like habitat preference. When analysing the connection between traits and extinction risk, our results suggest to not only consider phylogenetic influences, but differences between temporal and environmental units too.

## Supplementary Material

Supplementary figures and tables (Tietje and Rödel 2017)

## Supplementary Material

Lithology and specimen completeness references (Tietje and Rödel 2017)

## Supplementary Material

Paleobiology Database references (Tietje and Rödel 2017)

## Supplementary Material

R code and data files (Tietje and Rödel 2017)

## Data accessibility

R script and all data are available in the electronic supplementary material.
