# Supplementary material for: Contradicting habitat type-extinction risk relationships between living and fossil amphibians
Source: R Soc Open Sci. 2017 May 10;4(5):170051. doi: 10.1098/rsos.170051 (PMC5451811; doi:10.1098/rsos.170051)
Supplement: Supplementary figures and tables (Tietje and Rödel 2017) [file rsos170051supp1.doc]

**Contradicting habitat type - extinction risk relationships between living and fossil amphibians**

Melanie Tietje and Mark-Oliver Rödel

# Supplement

## Data files

- R script and data files: *Tietje_Rodel_Rscript_and_data_files_ESM.tar*
- Paleobiology Database download reference data: *Tietje_Rodel_pbdb_references_ESM.csv*
- Lithology and specimen completeness metric references: *Tietje_Rodel_lithology_and_SCM_references_ESM.csv*

## Tables

Table S1 Usage of taxonomic group names in the databases FosFAR (Database of Vertebrates: Fossil Fishes, Amphibians, Reptiles, Birds) and Paleobiology Database (PbDb) in this publication.

| **Taxon name used in this publication** | **FosFARbase** | **PbDb** | **Description** |
| --- | --- | --- | --- |
| Allocaudata | Allocaudata Fox&Naylor, 1982 | Albanerpetontidae  Fox&Naylor, 1982 | The included species are identical. PbDb does not use Allocaudata Fox & Naylor, 1982 as order though. |
| Species from stem groups were assigned to their corresponding pan-groups Salientia, Urodela and Parabatrachia | - | Amphibia Linnaeus, 1758 | Amphibia covers all stem group taxa in amphibians that were not covered by Anura, Caudata or Gymnophiona, it also includes Lepospondyli. This taxonomic rank is not available in FosFARbase. |
| Salientia. Includes Anura and their stem-taxa [1] | Anura  Rafinesque, 1815 | Anura  Fischer von Waldheim, 1813 | PbDb Anura contains crown-group Anura, FosFARbase Anura contains stem-group Anura like *Triadobatrachus massinoti* |
| Urodela. Includes all Caudata and their stem-taxa [1] | Caudata  Scopoli, 1777 | Caudata  Scopoli, 1777 | FosFARbase includes stem-taxa only, PbDb includes both stem- and crown-taxa. |
| Parabatrachia. Includes Gymnophiona and their stem-taxa [1] | Gymnophiona Rafinesque, 1814 | Gymnophiona Rafinesque, 1814 | Both databases include stem group taxa despite using the definition by Rafinesque 1814. |
| Lepospondyli | - | Lepospondyli, von Zittel 1887 | Lepospondyli were only available from PbDb, included in Amphibia |
| Temnospondyli. Without Lissamphibia[2] | Temnospondyli  Von Zittel, 1888 | Temnospondyli von Zittel, 1887 | - |
| Urodela. Includes Caudata and their stem-taxa [1] | Urodela  Latreille, 1825 | Urodela  Duméril, 1805 | Groups in both databases contain the crown-group Caudata. |

Table S2 Species name synonyms. We used PbDb names in our analysis.

| **PbDb name** | **fosFAR name** | **Reference** |
| --- | --- | --- |
| *Albanerpeton nexuosus* | *Albanerpeton nexuosum* | [3] |
| *Albanerpeton pannonicus* | *Albanerpeton pannonicum* | [3] |
| *Rewana myriadens* | *Arcadia myriadens* | [3] |
| *Koskinonodon perfectus* | *Buettneria howardensis* | [3] |
| *Koskinonodon perfectus* | *Buettneria perfecta* | [3] |
| *Gerrothorax pulcherrimus* | *Gerrothorax rhaeticus* | [3] |
| *Tatrasuchus wildi* | *Kupferzellia wildi* | [3] |
| *Palaeobatrachus tobieni* | *Messelobatrachus tobieni* | [3] |
| *Meyerosuchus fuerstenbergianus* | *Meyerosuchus fuerstenberganus* | [3] |
| *Scaphiopus alexanderi* | *Spea alexanderi* | [3] |
| *Spea pliobatrachus* | *Spea pliobatracha* | [3] |
| *Bufo defensor* | *Anaxyrus defensor* | [4] |
| *Bufo hibbardi* | *Anaxyrus hibbardi* |
| *Bufo repentinus* | *Anaxyrus repentinus* |
| *Bufo rexroadensis* | *Anaxyrus rexroadensis* |
| *Bufo tiheni* | *Anaxyrus tiheni* |
| *Bufo valentinensis* | *Anaxyrus valentinensis* |

Table S3 Completeness measure estimates for different taxonomic levels in amphibians. “No REP” indicates no range end points of species were included in the calculations.

| **Group** | **Proportion** |
| --- | --- |
| *Simple completeness metric (SCM)* |  |
| Species | 0.94 |
| Species no REP | 0.44 |
| Genera | 0.78 |
| Genera no REP | 0.35 |
| Families | 0.60 |
| Families no REP | 0.28 |
| Families Cretaceous | 0.43 |
| Families Cretaceous no REP | 0.30 |
| *Preservation probability based on duration frequency* |  |
| Species | 0.35 |
| Genera | 0.47 |
| *Proportion of living taxa with fossil record* |  |
| Families | 0.33 |
| Genera | 0.08 |
| Species | 0.008 |
| *Proportion of living taxa with fossil record for mammals* |  |
| Families | 0.69 |
| Genera | 0.03 |
| Species | 0.002 |

Table S4 Comparisons of specimen completeness in different habitat categories (level 1). Groups were *stagnant* (81), *low-velocity* (72), *medium-velocity* (35) and *high-velocity* (14). We used Kruskal-Wallis rank sum test (*χ²* (df), p-value) and Pairwise Wilcoxon Rank Sum Tests for pairwise comparisons (P-value adjustment: fdr). Significant p-values (*p*< 0.05) are shown in bold.

|  | Groups | | |
| --- | --- | --- | --- |
| *Kruskal-Wallis test* |  |  |  |
| χ² (df) | 18.03 (3) |  |  |
| p-value | **0.0004** |  |  |
| *Wilcoxon rank-sum test* |  |  |  |
|  | *Stagnant* | *Low-velocity* | *Medium-velocity* |
| *Low-velocity* | **0.0013** | - | - |
| *Medium-velocity* | **0.0222** | 0.8956 | - |
| *High-velocity* | 0.1369 | 0. 8956 | 0. 8956 |

Table S5 Comparisons of specimen completeness in different taxonomic groups. We used Kruskal-Wallis rank sum test (χ² (df), p-value) and Pairwise Wilcoxon Rank Sum Tests for pairwise comparisons (P-value adjustment: fdr). Significant p-values (p< 0.05) are shown in bold. Group sizes were: No-Lepospondyli (189), No-Temnospondyli (159), Lepospondyli (13), Temnospondyli (43).

|  | **Groups** | | |
| --- | --- | --- | --- |
| *Kruskal-Wallis test* | *Lissamphibia* | *Others* |  |
| χ² (df) | 6.54 (3) | 45.93 (3) |  |
| p-value | 0.09 | **< 0.0001** |  |
| *Wilcoxon rank-sum test* |  |  |  |
|  | No-Lepospondyli | No-Temnospondyli | Lepospondyli |
| No-Temnospondyli | 0.27 | - | - |
| Lepospondyli | **< 0.0001** | **< 0.0001** | - |
| Temnospondyli | **< 0.0001** | **< 0.0001** | 0.10 |

Table S 6 Comparison of species duration from different environments (for category definitions see Fig. 1). Groups were stagnant (214), low-velocity (130), medium-velocity (56) and high-velocity (18); lentic (216) and lotic (176); low (319) and high (71). We used Kruskal-Wallis rank sum test (χ² (df), p-value) and Pairwise Wilcoxon Rank Sum Tests for pairwise comparisons (p-value adjustment: fdr). Significant differences (p< 0.05) are given in bold.

|  | Level 1 | Level 2 | Level 3 | |
| --- | --- | --- | --- | --- |
| *Kruskal-Wallis test* |  |  |  | |
| χ² (df) | 17.86 (3) | 4.33 (1) | 17.27 (1) | |
| p-value | **0.0005** | **0.0375** | **<0.0001** | |
| *Wilcoxon rank-sum test for level 1* |  |  | |  |
|  | Stagnant | Low-velocity | | Medium-velocity |
| Low-velocity | 0.0568 | - | | - |
| Medium-velocity | **0.0001** | 0.1128 | | - |
| High-velocity | 0.2385 | 0.8187 | | 0.5071 |

Table S7 Comparisons of species duration between different taxonomic groups. Groups were Allocaudata (11), Urodela (39), Parabatrachia (2), Salientia (80) and No-Temnospondyli (171), No-Lepospondyli (310), Lepospondyli (39), Temnospondyli (178). We used Kruskal-Wallis rank sum test (χ ² (df), p-value) and Pairwise Wilcoxon Rank Sum Tests for pairwise comparisons (P-value adjustment: fdr). Significant p-values (p< 0.05) are shown in bold.

| **Lissamphibia group** |  | | |
| --- | --- | --- | --- |
| *Kruskal-Wallis test* |  | | |
| χ² (df) | 12.59 (3) |  |  |
| p-value | **0.0056** |  |  |
| *Wilcoxon rank-sum test* |  |  |  |
|  | Allocaudata | Parabatrachia | Salientia |
| Parabatrachia | 0.3569 | - | - |
| Salientia | **0.0059** | 0.5358 | - |
| Urodela | 0.1717 | 0.4638 | 0.1374 |
|  |  |  |  |
| **Other groups** |  |  |  |
| *Kruskal-Wallis test* |  |  |  |
| χ² (df) | 9.31 (3) |  |  |
| p-value | **0.0255** |  |  |
| *Wilcoxon rank-sum test* |  |  |  |
|  | No -Lepospondyli | No -Temnospondyli | Lepospondyli |
| No –Temnospondyli | 0.2640 | - | - |
| Lepospondyli | 0.3800 | 0.2100 | - |
| Temnospondyli | 0.1770 | **0.0240** | 0.9630 |

Table S8 Observed proportions divided by expected proportions of species occurrences in each environment and taxonomic group under the assumption of equal proportions distribution. Results were computed using Pearson’s chi-square test. Deviations larger than 20% are shown in bold.

|  | Stagnant | Low-velocity | Medium-velocity | High-velocity |
| --- | --- | --- | --- | --- |
| *Others* |  |  |  |  |
| No-Lepospondyli | 0.94 | 1.07 | 1.07 | 0.99 |
| No-Temnospondyli | 1.09 | 0.76 | **1.27** | **0.77** |
| Lepospondyli | **1.57** | **0.32** | 0.36 | 1.14 |
| Temnospondyli | 0.91 | **1.24** | **0.72** | 1.24 |

Table S9 Comparisons of durations of species from different environments and groups. We used Kruskal-Wallis rank sum test (χ² (df), p-value). Significant p-values (p< 0.05) are given in bold. Pairwise comparisons for groups with significant differences are displayed in Table S 10.

|  | *Level 1* | | *Level 2* | | *Level 3* | |
| --- | --- | --- | --- | --- | --- | --- |
| *Lissamphibia* | p-value | χ²(df) | p-value | χ² (df) | p-value | χ² (df) |
| Allocaudata | 0.7861 | 1.1 (3) | 0.7843 | 0.1 (1) | 0.8616 | 0.03 (1) |
| Salientia | **0.0025** | 14.3 (3) | **0.0082** | 6.9 (1) | **0.0234** | 5.14 (1) |
| Urodela | 0.0818 | 5.0 (2) | 0.1062 | 2.6 (1) | 0.1017 | 2.7 (1) |
| *Others* |  |  |  |  |  |  |
| No – Temnospondyli | **<0.0001** | 14.2 (3) | **0.0014** | 10.2 (1) | **0.0017** | 9.9 (1) |
| No – Lepospondyli | **0.0028** | 22.4 (3) | 0.0649 | 3.4 (1) | **0.0013** | 14.5 (1) |
| Lepospondyli | 0.3673 | 3.4 (3) | 0.9765 | 0 | 0.4559 | 0.6 (1) |
| Temnospondyli | 0.5034 | 2.3 (3) | 0.9754 | 0 | 0.0530 | 3.7 (1) |

Table S10 P-values for pairwise comparisons of durations in level 1 habitat categories in the groups Salientia, No-Lepospondyli and No-Temnospondyli. We used Pairwise Wilcoxon Rank Sum Tests with fdr p-value adjustment). Significant p-values (*p*< 0.05) are shown in bold.

|  | No -Lepospondyli | | No -Temnospondyli | Salientia |
| --- | --- | --- | --- | --- |
| Stagnant / low-velocity | 0.0859 | **0.0002** | | **0.0068** |
| Stagnant / medium-velocity | **0.0012** | **0.0002** | | **0.0032** |
| Stagnant / high-velocity | 0.2013 | 0.5459 | | 0.7064 |
| Low-velocity / medium-velocity | 0.2013 | 0.9849 | | 0.7064 |
| Low-velocity / high-velocity | 0.7021 | 0.5459 | | 0.4837 |
| Medium-velocity / high-velocity | 0.7021 | 0.5459 | | 0.4837 |

Table S11 Comparison of species geographic ranges from different environments (for category definitions see Fig. 1). Groups were stagnant (214), low-velocity (129), medium-velocity (56) and high-velocity (18); lentic (214) and lotic (175); low (318) and high (71). We used Kruskal-Wallis rank sum test (χ² (df), p-value) and Pairwise Wilcoxon Rank Sum Tests for pairwise comparisons (p-value adjustment: fdr). Significant differences (p< 0.05) are given in bold.

|  | Level 1 | Level 2 | Level 3 | |
| --- | --- | --- | --- | --- |
| *Kruskal-Wallis test* |  |  |  | |
| χ² (df) | 19.1 (3) | 0.42 (1) | 19.3 (1) | |
| p-value | 0.0003 | 0.5161 | **< 0.0001** | |
| *Wilcoxon rank-sum test for level 1* |  |  | |  |
|  | Stagnant | Low-velocity | | Medium-velocity |
| Low-velocity | 0.5858 | - | | - |
| Medium-velocity | **0.0242** | 0.0946 | | - |
| High-velocity | **0.0004** | **0.0027** | | 0.1519 |

Table S12 Comparison of species duration from different environments, controlled for geographic range. Groups were split according to the geographic range of the species being 1 or more grid cell counts. Groups for were stagnant (185|29), low-velocity (110|20), medium-velocity (41|15) and high-velocity (9|9); lentic (185|29) and lotic (148|28); low (284|35) and high (49|22). We used Kruskal-Wallis rank sum test (χ² (df), p-value) and Pairwise Wilcoxon Rank Sum Tests for pairwise comparisons (p-value adjustment: fdr). Significant differences (p< 0.05) are given in bold.

|  | Level 1 | | Level 2 | | Level 3 | |
| --- | --- | --- | --- | --- | --- | --- |
|  | 1 | >1 | 1 | >1 | 1 | >1 |
| *Kruskal-Wallis test* |  |  |  |  |  |  |
| χ² (df) | 14.58 (3) | 1.67(3) | 6.61 (1) | 0.002(1) | 6.51 (1) | 0.72(1) |
| p-value | **0.0022** | 0.6443 | **0.0176** | 0.967 | **0.0107** | 0.3958 |
| *Wilcoxon rank-sum test for level 1, range = 1* |  |  |  |  |  |  |
|  | Stagnant | Low-velocity | Medium-velocity |  |  |  |
| Low-velocity | 0.0576 | - | - |  |  |  |
| Medium-velocity | **0.0022** | 0.2244 | - |  |  |  |
| High-velocity | 0.3791 | 0.2244 | 0.1413 |  |  |  |

Table S13 Deviation of observed from expected proportions of single-interval species between energetic settings according to Pearson's chi-squared test.

| **Habitat** | **Single-interval** | **Longer** |
| --- | --- | --- |
| *Stagnant* | 1.08 | 0.70 |
| *Low-velocity* | 0.97 | 1.11 |
| *Medium-velocity* | 0.78 | 1.87 |
| *High-velocity* | 0.97 | 1.11 |

Table S 14 Comparison of species duration from different environments excluding single-interval species (for category definitions see Fig. 1). Groups were stagnant (30), low-velocity (29), medium-velocity (21) and high-velocity (4); lentic (30) and lotic (38); low (42) and high (24). We used Kruskal-Wallis rank sum test (χ² (df), p-value) and Pairwise Wilcoxon Rank Sum Tests for pairwise comparisons (p-value adjustment: fdr). Significant differences (p< 0.05) are given in bold.

|  | Level 1 | Level 2 | Level 3 |
| --- | --- | --- | --- |
| *Kruskal-Wallis test* |  |  |  |
| χ² (df) | 3.88 (3) | 1.06 (1) | 3.77 (1) |
| p-value | 0.1808 | 0.3039 | 0.0523 |

## Figures


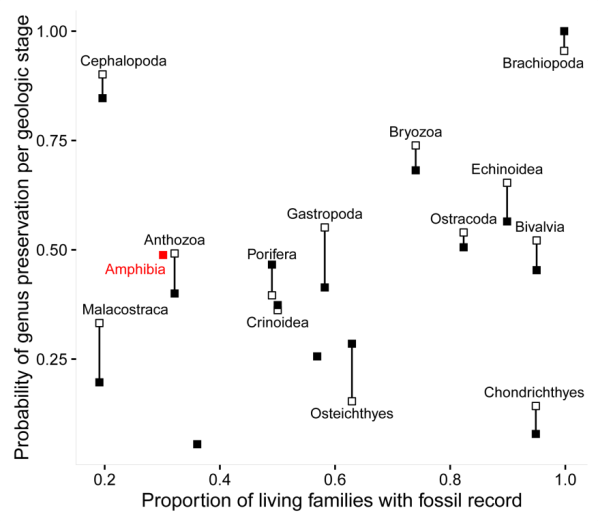


Figure S1 Proportion of living families with a fossil record plotted against probability of genus preservation (adapted from Figure 1 by Foote & Sepkoski [5]). The red dot shows values for our fossil amphibian dataset.


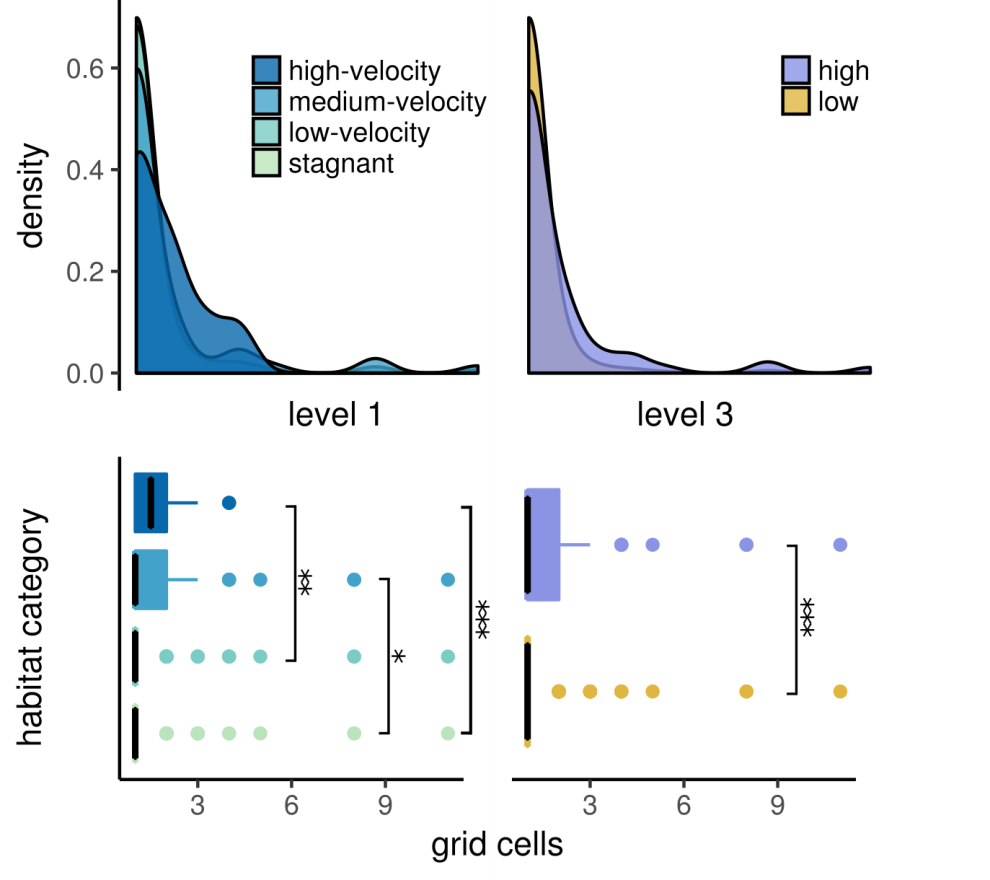


Figure S2 Geographic ranges in number of occupied grid cells for species in different environments. Categories and colour coding are as defined in Figure 1. Sample sizes for the groups are the same as in Figure 3. The upper panel shows the density distribution of durations (bandwidth=2 million years), the lower panel shows the durations as boxplots, with black lines indicating the median and coloured areas showing the range between first and third quartiles. Significance levels are indicated by one, two and three asterisks representing p-values smaller than 0.05, 0.01 and 0.001, respectively.

#
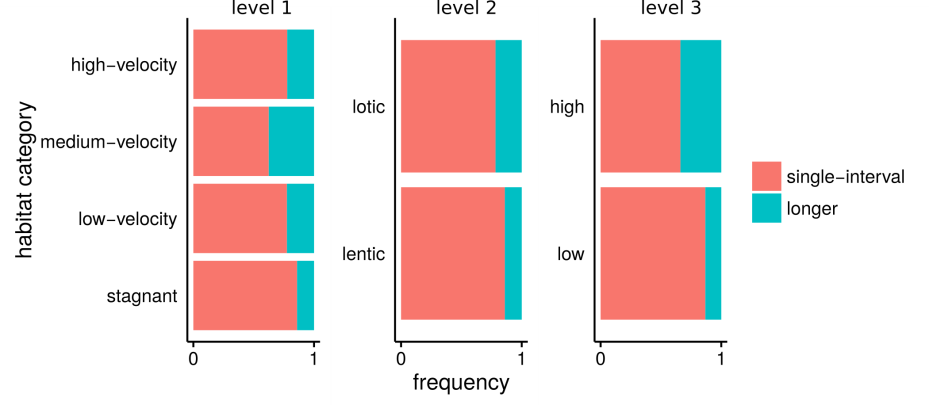


Figure S3 Frequencies of single-interval species in different habitat categories.


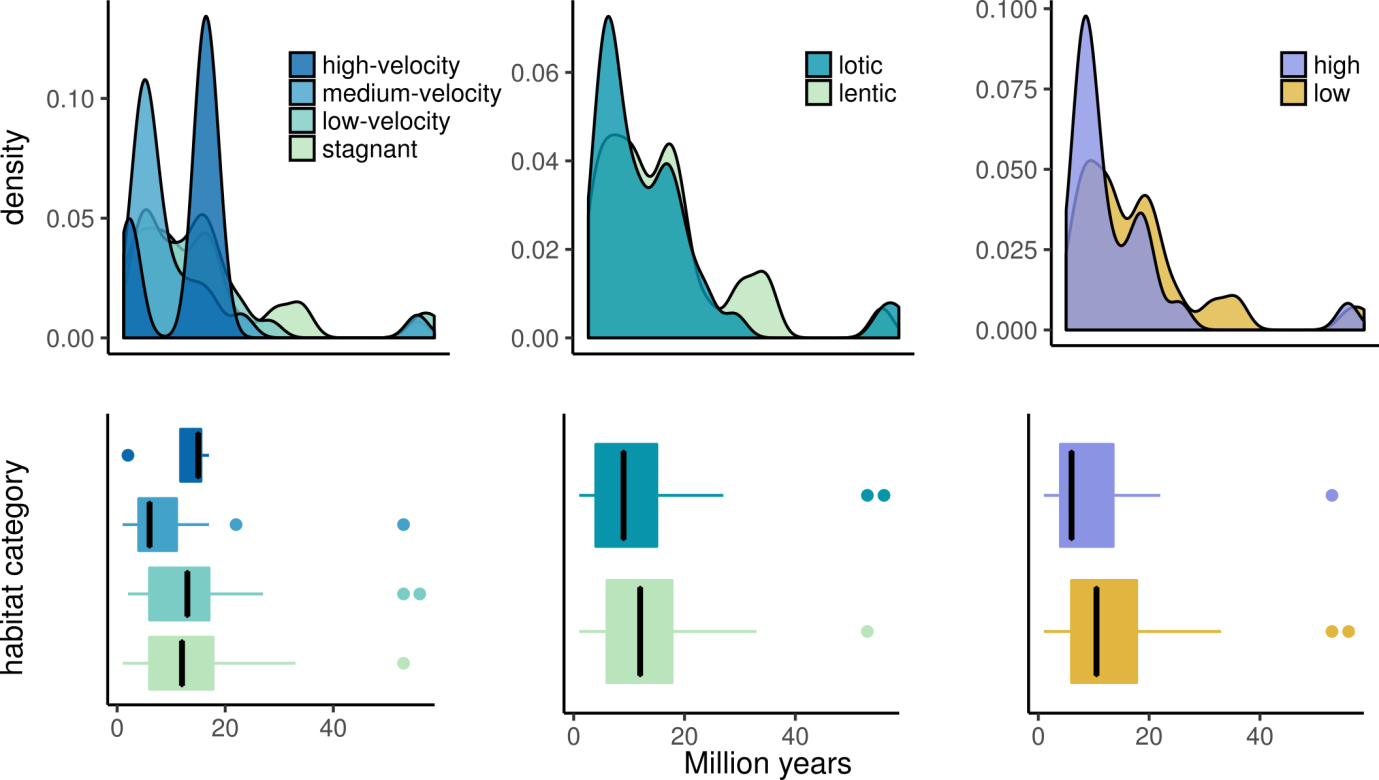


Figure S4 Durations of amphibian species excluding single-interval species in different environments. Species were grouped into four basic (level 1) and two broader environmental categories (level 2 and 3; compare Figure 1 in the manuscript. Sample sizes for groups were: stagnant (30), low-velocity (29), medium-velocity (21), high-velocity (4); lentic (30) and lotic (38); low (42) and high (24).

## References supplement

1. Frost DR et al. 2006 The Amphibian Tree of Life. *Bull. Am. Museum Nat. Hist.* **297**, 1–291. (doi:10.1206/0003-0090(2006)297[0001:TATOL]2.0.CO;2)
2. Schoch RR, Milner AR. 2014 Handbook of Paleoherpetology - Part 3A2: Temnospondyli I. Verlag Dr. Friedrich Pfeil, München
3. Fossilworks 2016. [http://fossilworks.org](http://fossilworks.org/)
4. Martín C, Alonso-Zarazaga MA, Sanchiz B. 2012 Nomenclatural notes on living and fossil amphibians. *Graellsia* **68**, 159–180. (doi:10.3989/graellsia.2012.v68.056)
5. Foote M, Raup DM. 1996 Fossil preservation and the stratigraphic ranges of taxa. *Paleobiology* **22**, 121–140. (doi:10.1017/S0094837300016134)
